# The Ways of Using Social Media for Health Promotion Among Adolescents: Qualitative Interview and Focus Group Study

**DOI:** 10.2196/71510

**Published:** 2025-06-09

**Authors:** Elizabeth Zimmermann, Samuel Tomczyk

**Affiliations:** 1 Department Health and Prevention Institute of Psychology University of Greifswald Greifswald Germany; 2 German Center for Child and Adolescent Health (DZKJ), partner site Greifswald/Rostock Greifswald Germany

**Keywords:** social media, intervention, features, adolescents, Instagram, health promotion

## Abstract

**Background:**

Social media offers promising, low-cost, and accessible ways to promote adolescent health within their daily routines. Platforms such as Instagram engage users through interactive features—including quizzes, question stickers, and polls—that encourage participation and behavior change. However, risks such as addiction potential and exposure to harmful content highlight the need for carefully designed interventions.

**Objective:**

This study aims to explore adolescents’ preferences and needs for Instagram-based health promotion to inform the design of our life skill intervention, “leduin (Lebenskompetent durch Instagram),” which means “life skills through Instagram.”

**Methods:**

Following a qualitative approach, we organized 12 semistructured interviews and focus groups with 67 adolescents aged between 14 and 17 years (women: n=37, 55%; men: n=29, 43%; and nonbinary: n=1, 2%), recruited via Instagram advertisements and from schools in Germany. We conducted 5 school-based focus groups (grammar school: n=1, 20%; Montessori school: n=2, 40%; and special education school: n=2, 40%) and 2 web-based focus groups. In addition, 5 individual interviews with boys were carried out to balance gender representation, as boys were less vocal in group settings. Data were analyzed using content analysis with a combined deductive-inductive coding approach in MAXQDA software, achieving high intercoder reliability (Cohen κ=0.93). The study design followed co-design principles, the social media uses and gratifications theory, and a cultural sensitivity framework.

**Results:**

Adolescents valued Instagram for social connection, personal growth, and engaging content, but some felt these programs might resemble schoolwork or attract only those already interested. To improve engagement, adolescents suggested combining intrinsic motivation (authentic connections) with extrinsic incentives, such as vouchers or praise. Adolescents recommended that health programs be visually engaging, interactive, and personalized, ideally featuring peers for better relatability. Posts and reels were popular for concise information, especially when using short, subtitled reels with engaging captions. However, the advertisements were disliked. Immersive, brief stories were appreciated, with interactive features, such as quizzes, polls, and question stickers, being valued for their brevity, curiosity appeal, and anonymity. Adolescents found live videos less appealing, citing logistical issues and a lack of structure, reflecting a preference for asynchronous content. Comments were rarely used due to privacy concerns, though some saw their potential for engaging with friends. Furthermore, most adolescents did not like notifications and switched them off.

**Conclusions:**

Findings support the social media uses and gratifications theory, highlighting the value of interactive and culturally sensitive content. While the small, region-specific sample limits generalizability, it reflects the depth of qualitative inquiry. Although social desirability and teacher presence may have influenced responses, efforts to foster openness aimed to minimize such effects. Instagram shows promise for adolescent health promotion through interactive, visually engaging, and culturally relevant content. Future research should explore broader and more diverse participant profiles to improve generalizability.

## Introduction

### Background

Social media is part of everyday life and the social environment of today’s adolescents [1] (adolescent is defined by the World Health Organization as a person aged between 10 and 19 years [2]). Moreover, 97% of US teenagers use the internet daily [3]. The frequency and duration of use grow with the progression of adolescence [3,4]. The EU Kids online survey results from 19 countries reveal that the amount of time spent online by adolescents aged between 15 and 16 years was nearly twice that of those aged between 9 and 11 years [5]. Social media is a place where young people engage with peers; formulate ideas; support each other; explore their identities; practice self-presentation; enhance autonomy, motivation, and decision-making capabilities; and share feelings, experiences, and problems [6-10]. At the same time, adolescents are also exposed to risks of social media, such as addiction, psychological impairment, fake news, cyberbullying, or harmful content [4,11-15], and they often lack social, emotional, and cognitive skills required for functional social media use, such as understanding interrelationships or underlying mechanisms, distinguishing between reputable and dubious sources, making moral decisions and acting accordingly, or making profitable use of social exchange [16-18].

Therefore, from the perspective of prevention and health promotion, social media is a double-edged sword. On the one hand, its ubiquity in everyday life promises great reach among young people, making it a powerful tool for health professionals. On the other hand, its many risks need to be considered, addressed, and counterbalanced in social media–based interventions. In line with the Ottawa Charter for Health Promotion [19], which highlights the importance of both creating supportive environments and developing personal skills, social media interventions should not only mitigate risks but also empower adolescents to make informed choices and adopt positive online behaviors.

A detailed discussion of the challenges and benefits of social media for adolescent health is beyond the scope of this paper. Instead, we aim to explore the role of social media in health promotion by using Instagram (Meta Platforms, Inc) as an example, which remains a highly used social media platform among youth. In general, compared to research on adults, research on social media–based health promotion for adolescents is scarce and lacks evidence [20,21], particularly concerning social media other than Facebook [22,23]. This study aims to explore adolescents’ perspectives on using social media for preventive purposes, investigating how Instagram interventions can be designed to align with their preferences and needs. This provides the foundation for developing an Instagram-based intervention that promotes life skills and responsible social media use in adolescents in order to leverage social media’s benefits to mitigate potential risks [24].

### Social Media and Health Promotion With Adolescents

A number of characteristics make social media well-suited for use in prevention efforts. First, the huge popularity of social media makes it a promising tool for promoting health among young people [25], which is the most active age group in the digital space; recent data show that 95% of teenagers aged between 13 and 17 years use YouTube, 67% use TikTok, and 62% use Instagram [3]. Second, social media can facilitate access to health care, especially for marginalized youth [26]. Third, interventions via social media are cost-effective, have a wide reach, and can be used regardless of social or demographic factors [7]. For example, a systematic review and meta-analysis found that recruiting participants via social media was nearly twice as effective as non–social media methods, for each dollar spent, and 66% more effective compared to other online methods [27]. Fourth, digital platforms not only offer flexible and continuous support but can also promote integration into daily routines, which can increase participant engagement and reduce dropout rates [28-31].

In this way, social media interventions have demonstrated measurable positive impacts on various health outcomes. In a systematic review, Guse et al [32] found that social media–based interventions significantly promoted safe sexual practices, such as increased condom use in adolescent populations, when compared to traditional methods. Similarly, Laranjo et al [33] conducted a meta-analysis that quantified the effect size for behavior change interventions on health outcomes through social media, reporting a small but significant overall effect size (Hedges *g*=0.24, 95% CI 0.04-0.43). These interventions were more effective than non–social media–based strategies in promoting behaviors, such as physical activity and healthy eating. A recent meta-analysis on skin health promotion highlights social media’s potential for effectively disseminating information, visualizing health-related topics, and encouraging positive behavior change [34].

To be efficacious, social media–based interventions should not just be passive (eg, viewing videos on YouTube or TikTok); they also require active participation. In this context, research highlights the importance of interactivity in digital learning. Interactivity significantly influences learning outcomes, with higher interactivity leading to better engagement and learning success [35]. Interactive activities foster a sense of connection between users and the program, which is crucial for engagement [36]. Integrating interactive features into interventions can thus reduce dropout rates and boost confidence to change [37]. Consequently, higher engagement levels are associated with greater symptomatic improvements [38]. Many features can be used to create interactivity and engagement. Social forums and peer interactivity are identified as key strategies to enhance user engagement [39-41]. Interactive gamification elements, such as challenges, points, incentives, and rewards, further support engagement and sustained use of health apps [40-46]. Well-designed program content; the use of multimedia formats and relevant links; and regular host activity, for example, by posing questions and providing supportive feedback, additionally support engagement [37,47]. Overall, maintaining active and interactive digital environments is crucial for the success of health promotion programs. Because not all social media platforms offer this variety of formats (eg, text-based platforms), some platforms, such as Instagram, might be more suited for interventive purposes.

### Interactive Social Media and Its Potential for Health Promotion

Due to its many interactive features, Instagram is increasingly being researched as a valuable platform for health promotion [48-51]. It offers various interactive elements, such as comments, likes, sliders, polls, quizzes, question boxes, direct messages, and quick reactions. These features result in 30 to 200 times higher interaction rates in health promotion campaigns on Instagram compared to Facebook or Twitter [52]. Instagram provides a feed and a story format. The latter offers content that disappears after 24 hours and can be designed with these interactive and entertaining features. However, the Instagram Story is a relatively new concept, and research on its potential for health promotion is scarce [53,54]. Some research has been conducted on the Snapchat Story feature, the format on which the Instagram Story feature is based [55]. However, these research results are only partly applicable to Instagram Stories, as they have evolved far beyond the possibilities of the original Snapchat Stories, offering a wider range of interactive and creative tools that have extended the original functionality. Unlike the original Snapchat Stories, which focused primarily on ephemeral content sharing, Instagram Stories allow users to add a variety of interactive features, such as polls, quizzes, shopping links, countdowns, and swipe-up links. Instagram also introduced the Highlights feature, enabling users to preserve their stories beyond the 24-hour limit, a feature not present in the original Snapchat format [56,57]. However, little is known about the preferences of the features available on Instagram, such as young people aged <18 years preferring video content over image and text content [48,58]. For successful health promotion among adolescents, further insights from adolescents themselves are needed.

Consequently, research has begun to explore Instagram’s potential for health promotion, highlighting the importance of using relatable visuals, authentic language, humor, and messaging that aligns with the audience’s cultural and social identities [59-61]. Accordingly, interventions that feature culturally resonant content can enhance engagement and retention in adolescent health campaigns. Malloy et al [62] emphasized addressing barriers, such as digital literacy and privacy concerns, to improve the effectiveness of social media interventions. Furthermore, incorporating interactive elements, such as polls and quizzes, made content more engaging and accessible. However, little is known about adolescent preferences for these features regarding health promotion and preventive interventions.

### Study Aims

To this end, this study aims to investigate the preferences and needs of adolescents regarding health promotion programs and preventive interventions on Instagram. Specifically, it seeks to identify design criteria, features, and approaches that resonate with adolescents while addressing their interests, motivations, and concerns. By exploring adolescents’ preferences and digital behaviors, this study aims to identify key factors that could enhance engagement in social media interventions, encourage positive online habits, and support their overall well-being in future social media interventions. In line with the Ottawa Charter for Health Promotion [19], which emphasizes enabling people to take control of their health by creating supportive environments and developing personal skills, this study aims to inform the development of low-threshold health promotion programs that function in the sense of universal prevention in reaching a broad audience. Furthermore, the findings were used to directly inform the development of an Instagram-based intervention by the authors, designed to promote life skills and responsible social media use, called “leduin (Lebenskompetent durch Instagram),” which means “life skills through Instagram.” Our program was designed to address the growing intersection of the analog and digital worlds by equipping adolescents with life skills that are applicable to both spheres. Life skills, as defined by the World Health Organization [63], are competencies that empower individuals to manage daily challenges effectively. Research has demonstrated that these skills not only enhance overall well-being and academic success but also reduce risky behaviors [64-68].

## Methods

This study was conducted in accordance with the American Psychological Association Style Journal Article Reporting Standards for Qualitative Research [69] and the Consolidated Criteria for Reporting Qualitative Research [70].

### Research Design, Theoretical Framework, and Rationale

This study used a qualitative design to enable an in-depth exploration of adolescents’ attitudes, preferences, and needs, crucial for developing tailored interventions. Using qualitative content analysis of interviews and focus groups, this approach allowed for detailed recommendations and a comprehensive discussion of the research questions. It is particularly well suited for examining complex behaviors, opinions, and underlying motivations within the target group [71].

**Figure 1:**
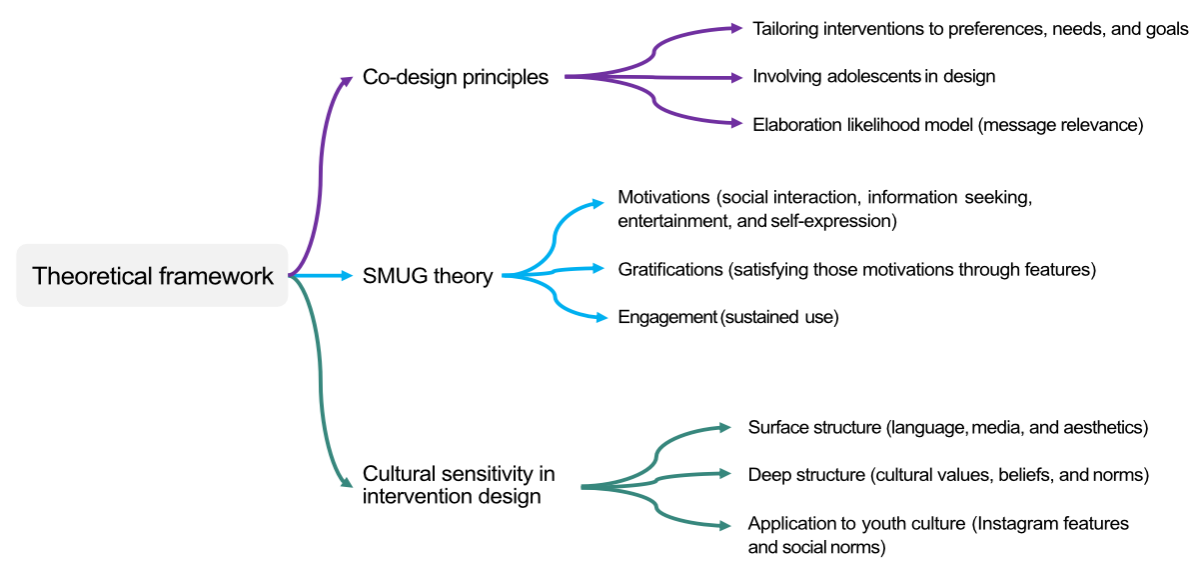
Theoretical framework for exploring adolescents’ preferences and needs regarding Instagram for health promotion. SMUG: social media uses and gratifications.

The qualitative content analysis was informed by the study’s theoretical framework that combined *co-design principles* [72], the *social media uses and gratifications (SMUG) theory* [73], and the *cultural sensitivity approach* to intervention design by Resnicow et al [74], as presented in Figure 1.

First, co-design principles emphasize tailoring interventions to the target group’s preferences, needs, and goals [72], with research showing that individualized approaches significantly improve engagement and behavior change outcomes [75,76]. This tailoring, based on the elaboration likelihood model [77], enhances message relevance, leading to increased attention and deeper processing [78]. Co-design facilitates this process by involving adolescents in the intervention design. In this study, adolescents provided input on their preferences regarding Instagram features and content for health promotion, ensuring that the intervention was relevant and engaging.

Second, the SMUG theory focuses on the motivations and gratifications that drive social media use, such as social interaction, information seeking, entertainment, and self-expression [73,79]. These motivations are satisfied through features such as messaging, commenting, and posting content, which can foster deeper user engagement when needs are met. In this study, the SMUG theory informed the creation of the interview guideline and the coding scheme by focusing on motivations (why users engage), gratifications (how features satisfy those motivations), and engagement (sustained use). Understanding these components is essential for designing interventions that align with adolescents’ specific social media behaviors.

Third, cultural sensitivity in intervention design involves tailoring both surface and deep structures of an intervention to the cultural characteristics of the target group [74]. Surface structure refers to visible aspects such as language, media content, and aesthetics that resonate with adolescents, while deep structure addresses underlying cultural values, beliefs, and norms that influence behavior. In this context, cultural sensitivity is applied to youth culture by integrating adolescents’ preferences for both observable Instagram features (surface structure) and their attitudes toward social media (deep structure). This dual approach ensures that the intervention is not only relevant to adolescents’ visible preferences but also resonates with their underlying social norms and behaviors.

In sum, these 3 theoretical concepts interlock to create a comprehensive framework that informs the study’s qualitative design. Co-design principles ensure that adolescents are actively involved in shaping the intervention, aligning it with their preferences and needs. The SMUG theory complements this by identifying key motivations and gratifications that drive adolescents’ social media engagement, enabling the intervention to effectively use Instagram’s features to capture their interest. Meanwhile, the cultural sensitivity approach ensures that both surface-level elements (eg, visuals and language) and deeper cultural values are integrated, enhancing the intervention’s relevance and resonance.

This study was grounded in a constructivist approach, acknowledging that adolescents construct their own meanings and understandings based on their interactions with Instagram [80,81]. An interpretive approach further helped to delve deeply into these meanings [82]. This combination allowed a comprehensive understanding of how adolescents perceived and interacted with Instagram features for health promotion. The qualitative design was particularly suitable for this study as it aimed to uncover the subjective experiences and perspectives of adolescents regarding Instagram’s features and their potential for health promotion.

### Ethical Considerations

The research project received approval from the ethics committee at the University Medicine Greifswald (BB 073/21). The approved procedures included conducting interviews and focus groups with adolescents to explore social media’s potential for health promotion.

All participants and their caregivers were fully informed about the study’s aims, methods, and data protection measures. To ensure voluntary participation, online participants and their caregivers provided informed consent via a secure, General Data Protection Regulation–compliant online survey platform designed for academic research, ensuring encrypted data transfer and secure storage. Students and their caregivers signed and returned the consent forms in analog form before the interviews and focus groups took place. In school settings, only those students who returned consent forms actively participated, while the other students remained in the room for logistical reasons and participated passively as they were part of the class.

All data were collected and stored in accordance with German data protection regulations. Data were anonymized to maintain participant confidentiality. No identifying details are included in this paper, and participants’ anonymity was ensured throughout the research process.

Participants received no compensation or incentives for their participation in this study. Recruitment focused on voluntary involvement to ensure authentic engagement with the topic.

No images of participants were taken and are therefore not included in this paper or in Multimedia Appendix 1. Therefore, no additional image consent was required.

This study carefully addressed ethical considerations, particularly ensuring that participants’ voices were respected and their privacy protected. One ethical challenge was balancing the exploration of social media’s potential for health promotion with the acknowledgment of its risks, such as exposure to harmful content or cyberbullying. The researchers addressed this by encouraging participants to discuss both positive and negative aspects of Instagram, ensuring a balanced perspective.

### Research Flow and Team

The interviews and focus groups were conducted between January and February 2022 by author EZ and a team of 4 researchers with backgrounds in psychology. The team comprised 3 undergraduate students and 1 individual with a Bachelor’s degree. All researchers were affiliated with the University of Greifswald and were part of a research group focused on social media’s potential in health care. Accordingly, there was a previous understanding of the potential role of social media in health care that enhanced the data collection process, in that the interviewers could give explanations and further information to participants. The interview team consisted of 3 women and 1 man, all trained to conduct the interviews and focus groups. There were no preexisting relationships between the researchers and participants, ensuring unbiased data collection.

### Recruitment and Demographics

Participants for this study were selected using a combination of purposive and convenience sampling to ensure diversity and accessibility. Adolescents aged between 14 and 17 years were recruited via Instagram advertisements across Germany, while students in the 9th and 10th grades were recruited from schools in Mecklenburg–Western Pomerania and Berlin. This demographic was chosen because it represented a critical transformational phase where social norms strongly influenced behavior, making life skills interventions particularly impactful. During this period, adolescents form health behaviors that often persist into adulthood, underscoring the importance of reaching this group [83-85]. In addition, these grades serve as preparatory stages for transitioning to secondary school or graduating from school in Germany, further emphasizing their relevance for intervention efforts [85]. Given Instagram’s widespread popularity among this age group, it was considered an effective platform for both recruitment and intervention delivery. Schools were contacted via phone and email to identify classes interested in participating in the discussions. In this study, focus groups were integrated into a prevention unit.

While we aimed to include a diverse range of adolescents to capture varying perspectives on social media use and health promotion needs, participants had to be aged ≥14 years to ensure they could adequately understand the potential benefits and risks of social media. In addition, proficiency in German was required (as an inclusion criterion).

We conducted 5 focus groups in schools: 1 (20%) at a grammar school; 2 (40%) at a Montessori school; and 2 (40%) at a special education school (Förderschule), which provides targeted support for children and adolescents with special educational needs. Two web-based focus groups complemented this. On the one hand, these formats allowed us to reach a broad audience, as our aim was to collect insights from adolescents with diverse backgrounds within one age group. On the other hand, the focus groups allowed group discussions, which provided valuable collective insights. However, we observed that girls were more vocal in these settings, while boys participated less actively. To balance the gender representation and ensure a more comprehensive understanding of both perspectives, we complemented the focus groups with 5 individual interviews targeting boys. The characteristics of focus groups and interviews and their participants can be found in Table 1.

**Table 1:** Characteristics of focus groups and interviews (N=67).

Settings and code			Participants
**Focus group in school**			
	S01	Grammar school students in the 10th grade 10 women and 3 men	
	S02 and S03	Montessori school students in the ninth and 10th grades 15 women, 12 men, and 1 nonbinary	
	S04 and S05	Special education school students in the ninth grade 5 women and 8 men	
**Online focus group**			
	G01	Students from the ninth and 10th grades 6 women	
	G02	Students from the 10th grade 1 woman and 1 man	
**Online interview**			
	P01-P05	Students from the ninth, 10th, and 11th grades 5 men	

In total, 12 interviews and focus groups were conducted, forming the basis for the qualitative analysis. This number was estimated to be sufficient in order to reflect diverse perspectives, especially as the educational background and the composition of the interviews and focus groups were very heterogeneous. Consequently, informed consent was collected from 67 participants and their parents. We did not collect any data about those who refused to participate.

### Data Collection

Semistructured interviews and focus groups were conducted. This interview style provided a versatile approach that balanced the flexibility of unstructured interviews with the guidance of structured ones [86]. The format allowed researchers to dive deeply into the respondents’ experiences and perceptions while ensuring that essential topics were covered. This balance enabled the collection of rich, detailed data and the ability to explore new themes as they emerged during the conversation. Interviews were specifically conducted to complement the focus groups, particularly after observing that certain participants, especially boys, were less vocal in group settings. These interviews allowed us to capture more individual perspectives and balance gender representation in the data.

Gill et al [87] highlighted the strengths of semistructured interviews in qualitative research. They pointed out that this method facilitated a more relaxed and natural interaction between the interviewer and the participant, which can lead to more authentic and comprehensive responses. The authors also noted that semistructured interviews were particularly effective for exploring complex issues, as they allowed the interviewer to clarify responses and delve deeper into the participant’s thoughts and feelings.

The interview guidelines were developed by the research team following the recommendations for qualitative interviews with adolescents [88] and based on the current research on social media use as demonstrated in the theoretical framework of this study. Accordingly, the use of Instagram for health promotion and prevention purposes was discussed. The aim was to explore possibilities and challenges with a focus on the supporting role social media could play in health promotion, as opposed to exploring why it is not suitable. As the focus groups in schools were embedded in a prevention unit, adolescents were given information about social media, and they compiled the benefits, challenges, and risks of using social media. Life skills were then presented as a strategy for coping with the challenges of everyday life and social media use. Adolescents were then introduced to the concept of the planned Instagram-based life skill intervention, designed to promote a range of essential skills. These included personal skills, such as goal setting, decision-making, and managing emotions and stress; social skills, such as communication, empathy, meeting needs, and setting boundaries; and health-related skills, such as recognizing dangers, addressing addictive behaviors, handling digital violence, and evaluating information. Building on this information, adolescents were asked for their perspectives on Instagram in general and its use for life skill promotion in particular. The primary questions focused on the types of Instagram content that adolescents preferred, how they interacted with this content, and which formats (posts, stories, reels, and lives) they evaluated as particularly appealing or not. In addition, the discussions explored under what circumstances participants would comment on or engage with content. In doing so, the questions aimed to explore surface as well as deep structure variables [74] and, by that, get insights into the needs, gratifications, and motivations of using Instagram [73]. Furthermore, questions were raised about the design of a program aimed at teaching life skills through Instagram. Participants were asked how such a program should be structured to ensure that they and their friends would participate, and which incentives could encourage their involvement. The idea of challenges to promote interaction was also discussed, along with suggestions for appropriate prizes for the challenges.

During the online video meetings, participants joined from their homes, with only the participant and 1 interviewer present to ensure a private and comfortable setting. For the class discussions, 2 interviewers conducted the sessions, allowing one to take notes and ensure adherence to the protocol. To create a safe environment, it was crucial to have a teacher present, as the research team was completely unfamiliar with the students.

Reflexivity was a key focus for the research team both during interview training and throughout the discussions. We addressed potential biases, particularly the inclination to highlight the positive potential of social media for health care. This was openly discussed with the adolescents, who were consistently reminded that critical opinions and opposing views were equally valuable. Consequently, this study thoroughly considered the challenges related to using social media for prevention, aiming for a balanced perspective.

To encourage open and authentic responses, we deliberately created a safe and nonjudgmental environment during the interviews. Participants were reassured that there were no right or wrong answers, and their contributions were actively validated to foster trust and reduce anxiety, particularly when discussing sensitive topics. In doing so, we followed methodical approaches to interviewing in social work by Widulle [89] and the recommendations for qualitative interviews with adolescents [88] by warming up; listening attentively; shaping the relationship constructively; conveying acceptance and appreciation; communicating in an empathetic, understanding, and relationship-sensitive manner; mirroring statements; and, if necessary, moderating openly. At the same time, when exploring participants’ social media use and platform features, the interviewers maintained a neutral stance, used open-ended questions, and encouraged diverse perspectives to ensure objectivity and minimize potential bias. This balanced approach was essential for gathering both personal insights and practical suggestions for intervention design.

Interviews and focus groups were recorded and transcribed according to the guidelines by Dresing and Pehl [90]. Due to logistical considerations, transcripts were not returned to participants for feedback.

### Data Analysis

**Figure 2:**
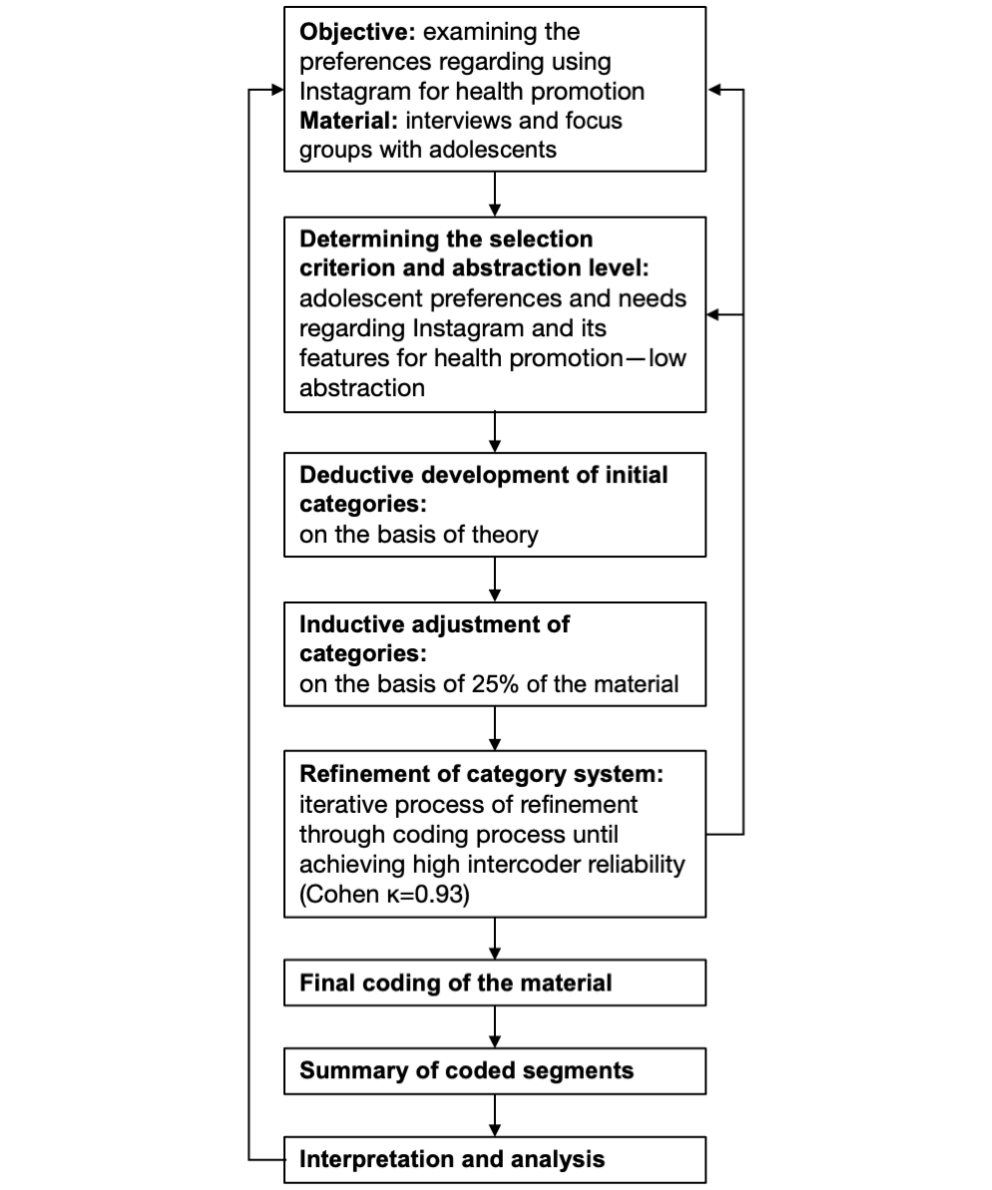
Process of qualitative content analysis on adolescent preferences for Instagram and its features.

This study used qualitative content analysis following the methods of Mayring and Fenzl [91], as presented in Figure 2, with the support of MAXQDA software (VERBI Software) for coding and data management [92]. Author EZ and another researcher (Simon Barth) served as data coders. A deductive-inductive approach was used, beginning with preexisting theories and research and refining categories based on the data collected from adolescents regarding Instagram’s use in health promotion. The analysis involved specifying selection criteria and maintaining a low abstraction level to capture detailed insights into adolescents’ interactions with Instagram. Interviews and focus groups served as units of analysis, and initial categories were developed based on theoretical frameworks. These were refined inductively after screening 25% of the data, leading to the creation of subcategories to better structure the adolescent statements. A category system, including definitions and anchor examples, was established to ensure consistent and reliable coding, with a Cohen κ of 0.93, indicating strong intercoder reliability [93]. An example of a category, subcategories, definitions, and anchor examples can be found in Table 2. The coding system used for the qualitative content analysis is detailed in Multimedia Appendix 1. Summarized results were then compared with research questions to confirm alignment with the study’s objectives, revealing detailed insights into adolescents’ preferences for health promotion on Instagram. Due to logistical constraints, participants were unable to provide feedback on the results.

**Table 2:** Example of the category system for the category “reasons for using Instagram.”

Subcategories	Definition	Anchor examples
General factors	General factors that could motivate adolescents to use Instagram for health promotion	“I think a lot of people would develop an interest in it and things like that.”
Social factors	Social variables that could motivate adolescents to use Instagram for health promotion	“I can teach you how to manage relationships better.”
Personal factors	Personal factors that could motivate adolescents to use Instagram for health promotion	“...because I’m also aware of some weaknesses where that would also help me.”

## Results

Overall, 67 participants contributed to the discussions, of whom 37 (55%) identified as women, 29 (43%) as men, and 1 (2%) as nonbinary. The focus groups and interviews lasted for an average duration of 62.5 (SD 29.9) minutes and a median of 75 (range 30-90) minutes.

### Final Category System

The final category system consisted of 1 system for each research question. The first system reflected general reasons for and barriers to using Instagram for health promotion as well as factors enhancing motivation in program participation, recommendations for program design, and diversity and identification potential. The second category system, which reflected the responses regarding the specific Instagram features, broke down the relevant statements by rating and frequency of use, reasons for and barriers to use, and positive and negative design for each feature individually. On the basis of the findings of this study, we developed a life skills prevention program called leduin (Lebenskompetent durch Instagram, meaning life skills through Instagram), delivered via Instagram. The program was first pilot-tested in a feasibility study to examine the use of Instagram as a delivery platform, and subsequently evaluated in a reachability study to explore factors influencing adolescents' initial engagement with the intervention [94].

### How Do Adolescents Evaluate the Possibilities of Prevention Efforts on Instagram in General?

#### Overview

The first research question concerned reasons for using Instagram for prevention and health promotion from the perspective of adolescents. Adolescents generally liked the idea of using Instagram for health promotion and highlighted several possible key reasons for using the platform for prevention efforts, which were grouped into 5 categories (Table 3). One participant remarked, “So in itself, the idea is really good and I think a lot of people would develop an interest in it and things like that” (G01).

**Table 3:** Summary of adolescents’ evaluation of Instagram’s (Meta Platforms, Inc) potential for health promotion.

Category	Key insights
Reasons for using Instagram	Adolescents appreciated Instagram for health promotion, citing improved social relationships, personal development, and relevance of content as key motivators.
Barriers to participation	Concerns included the program feeling like schoolwork and its limited appeal to only those already interested in the subject matter.
Factors enhancing motivation	Intrinsic motivation was boosted by personal connections and authenticity, while extrinsic motivation was enhanced by rewards, such as vouchers or praise.
Recommendations for program design	Programs should be visually engaging, interactive, easy to use, and accessible in long term. Simplicity and personal relevance are key.
Diversity and identification potential	Featuring diverse individuals, including real adolescents, improved relatability and identification, enhancing program effectiveness.

#### Reasons for Using Instagram

First, participants noted that the use of social media could improve social relationships and help build new contacts with other program participants. One individual shared the following:

A lot of people have probably had bad experiences in relationships and then you can probably bring in the aspect that you can say, I can teach you how to manage relationships better without paying for expensive therapy, I don’t know.

P01

Another participant added the following:

So I’m also quite convinced that you would do something like that, because it would be really useful to build up your social contacts a bit, because the corona situation is ruining everything and that would be a solution to find yourself better again.

G02

In addition, participants recognized the platform’s potential for personal development, including working on individual weaknesses and achieving better grades in school. One participant expressed, “Because I’m also aware of some weaknesses where that would also help me” (S02). The high personal relevance of a program was also rated as a very important motivator. Another participant stated the following:

It’s similar for me. If the things really appeal to me now and I think to myself “ok, I could really learn something there; that would help me,” I would look at it and read through it further.

S02

#### Barriers to Participation

The focus groups also identified barriers to participation in potential prevention programs on social media. Adolescents expressed concerns that the program might feel like extra schoolwork, which could be discouraging. One participant shared their hesitation, stating, “I think that’s something I wouldn’t want, so I don’t know if I’d feel like I’m still at school” (S01).

In addition, there was a worry that only those already interested in the subject matter would participate, potentially limiting the program’s reach and impact. One adolescent pointed out, “These are still just people who generally like this kind of thing and are interested in it” (S01).

#### Factors Enhancing Intrinsic and Extrinsic Motivation

Adolescents emphasized the importance of feeling a personal connection with the account manager in any social media–based prevention program. One participant expressed the following:

I think this idea of a personal bond is quite good. That there is someone you can always write to.

G01

They suggested that communication with a sympathetic, authentic person who shares personal experiences could significantly boost motivation. One adolescent noted, “Authenticity is important, so that they also come across naturally” (S02). Transparent communication in a relatable, conversational tone was also assessed as crucial, as adolescents wanted to feel respected and not lectured. One participant explained, “That there is this certain transparency for the audience, um, so that it doesn’t feel like you’re being lectured to again” (S01). Another added, “So for me it depends on the person, whether they are likeable” (S03).

Adolescents rated monetary rewards, such as gift vouchers or funds for the class, as effective means to potentially increase extrinsic motivation. They also found experiential rewards, such as cinema tickets or class activities, to be particularly motivating. In addition, praise and positive reinforcement were noted as strong motivators. One participant succinctly captured the sentiment, saying, “Well, you would do it if there was a reward” (P02). Another agreed, stating, “Yes, with a prize, I think you can attract a lot of people” (P03).

The preference for vouchers was particularly emphasized, as one adolescent explained the following:

Well, I would also say vouchers. That would be more interesting. It’s stupid when something special is raffled off that I or you don’t fancy. Vouchers are a bit more neutral and therefore better.

S03

#### Recommendations for Program Design

Adolescents provided several recommendations for the design of prevention programs on Instagram, emphasizing the importance of an engaging and intuitive layout with high visual quality that conveyed a serious impression. One participant suggested the following:

That you can also, uh I don’t know, assign themes or color or shapes or I don’t know, something that you know roughly on the channel, so you can recognize the structure and, uh, know what, so what belongs to what.

S01

Another added the importance of “good quality, good material” (S01). The seriousness of the content was also a key factor, as one adolescent mentioned the following:

I also have the feeling, especially with something like this, that it’s important that the account also radiates seriousness, that I really have the feeling, ok the things are really well researched, I can believe it now.

S01

Simplicity paired with high interactivity was found to be very important. One participant expressed the following:

I would make it very interactive, so that you can do things yourself, because it’s simply more interesting than just reading through posts.

S02

However, the program should not be overly complicated, as another participant pointed out the following:

But it shouldn’t be too difficult in any way, that you have to register for ages, I think a lot of people would drop out. So just simple things that are easy for everyone to do in a story or something.

P03

This simplicity was echoed by another adolescent, who said the following:

Well, that the whole topic is easy to grasp and you don’t have to really...I don’t want to say “read in for a long time,” but that you can grasp the whole topic quickly.

S02

Content should be available long-term rather than self-deleting to ensure that adolescents can come back to it. One participant emphasized, “And not then take it down or something. And that you can still access [previous modules]” (P02). The inclusion of different features, such as stories, polls, and quizzes, was also recommended to keep the program dynamic and interesting. To make the content more relatable and engaging, participants suggested including personal anecdotes and humorous elements and covering a variety of topics. One adolescent summarized, “It just has to have a lot of fun aspects somehow” (S01).

#### Diversity and Identification Potential

It was highlighted that the program should feature a diverse range of individuals, including the adolescents themselves, to create relatable and inclusive content. One participant suggested, “I would perhaps also include pupils or younger people from time to time...who you might interview” (P03). Another participant stated the following:

So if I have a person that I can identify with or a person that I can say “Oh, that person is really cool, I really like them” or something like that, but if only the uniform groups of people are represented, then I imagine it would be difficult.

S01

Accordingly, using real people with whom adolescents can identify, rather than models, was seen as crucial for improving identification and connection. One participant put it simply, saying, “Not just pictures of models.” (S02)

### How Do Adolescents Evaluate the Various Instagram Features for the Purpose of Prevention Efforts?

#### Overview

The second research question addressed specific Instagram features and how they were evaluated by adolescents for their potential use in prevention efforts. The research team discussed available interactive elements on Instagram to ensure a comprehensive assessment. The features examined included traditional posts (posts and reels), text, stories, live sessions, comments and comment reading, interactive story features (quizzes, polls, and question stickers), and notifications. Participants’ responses were coded in accordance with these features into 6 categories (Table 4).

**Table 4:** Summary of adolescents’ evaluation of Instagram features for prevention efforts.

Feature	Evaluation by adolescents
Posts and reels	Favored for quick information consumption Adolescents appreciated short, subtitled reels and engaging captions Advertisements were disliked
Stories	Viewed positively due to immersive experience and FOMO^a^ Appreciated when short and interactive Adolescents disliked too many slides
Interactive story features	Highly favored for brevity, curiosity, and social comparison Anonymity and simplicity were crucial.
Live sessions	Generally disliked due to logistical challenges and a lack of structure Adolescents preferred asynchronous content
Commenting and reading comments	Rarely used, mainly for friends’ posts Privacy concerns limited commenting, but high interest may drive engagement
Notifications	Viewed negatively Notifications disabled by most adolescents due to intrusiveness.

^a^FOMO: fear of missing out.

#### Posts and Reels

Adolescents highly favored traditional posts and reels on Instagram due to the ease of quick information consumption. They particularly appreciated posts with multiple images that allowed sideways scrolling, which provided a way to delve deeper into the content. Reels, especially those that were short (30 s to 1 min), informative, and subtitled, were especially popular among them. One participant noted, “I have to say, as soon as the videos are a bit longer, I start to switch off at some point” (S01). Another one stated, “For example, if you use a video or audio, you should always have subtitles” (P04).

An engaging caption was also seen as a crucial element, encouraging further reading and interaction. One adolescent mentioned, “If there is a caption under the article and it appeals to you, then you look more at the comments” (S02). However, advertisements within posts were negatively perceived by the adolescents, indicating a preference for content that felt genuine and free from commercial interruptions.

#### Stories

Adolescents evaluated stories positively for providing a more immersive experience compared to posts in the feed. One participant mentioned, “The stories from the accounts that I interact with the most, that I look at the most, I see them every day” (P04). Another highlighted how stories enhanced the quality of the experience, stating, “Then it has a stronger quality of experience because of the music and emotions” (S02).

They noted that the ephemeral nature of stories, which disappeared after 24 hours, created a fear of missing out, driving engagement. Stories were appreciated for facilitating quick information intake with minimal effort, as they automatically advanced. Adolescents recommended keeping stories short and limiting the number of slides per day, as one participant expressed, “I just don’t like it when the stories are really long, so many little ones in a row, I hate that like the plague and I always skip on” (G02).

Interactive elements within stories, such as polls and quizzes, were particularly well received. One adolescent shared, “Um, I also really like this interaction in stories” (S01).

#### Interactive Story Features

Quizzes, polls, and question stickers within stories were among the most favored features, according to the adolescents. One participant expressed their enthusiasm saying the following:

I actually think the quizzes are always really cool. You just have a quick look, click somewhere on what you think and you actually do that a lot.

P03

They enjoyed these elements for their brevity, ability to spark curiosity, and the opportunity for social comparison when they could see how others had responded. One adolescent noted, “Then you also see the opinions of other people, I find that really interesting” (S01).

Simplicity and the ease of participation were crucial, with a preference for clicking or providing short answers. Anonymity in responses was highly valued, allowing for candid participation. One participant suggested, “And that maybe you share them again anonymously at the end to somehow give the others an insight” (G01).

#### Live Sessions

Live sessions were generally not favored by adolescents, mainly due to logistical challenges. One participant explained, “When you come in somewhere in between, you always don’t know what it’s really about” (P01). They found that live sessions required real-time participation, which conflicted with Instagram’s asynchronous benefits, as another participant stated, “I don’t take extra time for this” (G01).

In addition, live sessions were often perceived as boring, and the live chat feature was therefore rarely used. One adolescent pointed out, “But quite often it’s just that it’s also super unstructured” (G01). Adolescents suggested that if live sessions are to be included in prevention programs, they should be well promoted in advance with clear scheduling and countdowns.

#### Commenting and Reading Comments

Most adolescents rarely commented on posts, and when they did, it was typically out of courtesy to friends. One participant plainly stated, “So I never actually comment” (S01). Another added, “Well, I only comment on my friends’ posts because then I can build them up a bit” (G01).

They mentioned that commenting might be considered if it allows active participation in shaping future content. However, concerns about privacy and the visibility of comments to others were significant barriers, particularly for sensitive topics. Comments were also infrequently read unless there was a high interest in others’ opinions. One adolescent explained the following:

Well, I tend to look at the comments when I’m interested in the topic and I think to myself “ok, I’m interested in what other people think about it and whether I’m not the only one with my opinion.”

S02

#### Notifications

Notifications were generally viewed negatively by adolescents, with many already having disabled Instagram notifications. They were seen as intrusive and not conducive to engagement. One participant stated the following:

I have the notifications off everywhere except WhatsApp. It gets on my nerves.

S02

## Discussion

### Principal Findings

This study explored how adolescents perceive the potential of Instagram and its features for health promotion and prevention efforts using qualitative research methods. Adolescents see potential in using Instagram for prevention efforts due to its capacity to improve social connections and personal development. They highlighted the importance of personal relevance but also noted barriers, such as the fear of it feeling like extra schoolwork, along with the risk of only attracting those already interested. According to adolescents, motivation to participate could be enhanced by fostering personal connections with relatable account holders; offering monetary and experiential rewards; and ensuring transparent, authentic communication. Adolescents emphasized the need for visually engaging, intuitive, and interactive program designs that are simple and long lasting.

Adolescents favored traditional posts and reels for quick information. They appreciated engaging captions and the visual appeal of reels, while advertisements were negatively perceived. Stories were also well received for their immersive and ephemeral nature, especially when interactive elements, such as quizzes and polls, were included. However, live sessions were generally rather disliked due to logistical challenges. Furthermore, adolescents only rarely comment, often limiting this social media activity to friends’ posts, with privacy concerns hindering more active participation. Notifications were viewed negatively, as most adolescents found them intrusive.

### Enhancing Adolescent Engagement: How Interactive Instagram Features and Cultural Sensitivity Drive Effective Health Promotion

The findings of this study align closely with the SMUG theory, which emphasizes that users engage with social media platforms to fulfill specific needs, such as social interaction, information seeking, entertainment, and self-expression [73]. Accordingly, in this study, adolescents highlighted these gratifications, particularly focusing on the importance of social interaction and personal development when engaging with health promotion content on Instagram. This aligns with broader research that consistently identifies social interaction as a primary driver of social media use among adolescents [9,10].

The study also applies the SMUG theory by identifying specific Instagram features (eg, quizzes and polls) that potentially meet these needs as reported by participating adolescents. Previous research, such as that by Edney et al [52], has shown that higher interactivity in digital learning programs enhances engagement. This study reinforces that conclusion by demonstrating adolescents’ preference for these interactive elements. In alignment with the findings that user engagement plays a critical role in the success of online interventions [35,36], this study demonstrates which features can best be used to support user engagement by meeting adolescents’ specific needs.

While much of the existing research has focused on the risks associated with social media use among adolescents, such as addiction, cyberbullying, and exposure to harmful content [4,12,13], this study contributes to the literature on the positive potential of social media for health promotion, a gap that has been identified before [95]. The findings support Dudley et al [25], who argue that social media platforms present unique opportunities for health promotion due to their widespread use, ability to reach diverse audiences, and integration into daily habits. As suggested in this study, content that is interacted with regularly will be shown in the user’s story and feed, thereby reaching adolescents during their daily use. This study identified several mechanisms regarding how to promote this interaction.

This study also underscores the relevance of the framework by Resnicow et al [74] on cultural sensitivity in health interventions, which advocates for both surface and deep structure tailoring. Adolescents in this study expressed a strong preference for relatable content and diverse representation within health promotion programs, reflecting the importance of surface structure tailoring. This finding aligns with the research by Johnson and Delk [59], which emphasizes the need for interventions that resonate with the cultural and social identities of the target audience. In addition, the adolescents’ desire for authentic and diverse representation indicates the importance of deep structure tailoring, where the intervention reflects the underlying cultural, social, and psychological factors that influence behavior.

### Balancing Interactive Features With the Need for Entertaining, Flexible, and Nonburdensome Content

The findings of this study have significant implications for the use of Instagram in adolescent health promotion. The strong preference for interactive and engaging content, such as quizzes, polls, and stories, indicates that these features are crucial for capturing and maintaining adolescents’ attention. However, this study also highlights significant barriers to participation, such as the perception that health promotion programs might feel like additional schoolwork. This finding is critical as it suggests that even well-designed programs may fail to engage adolescents if they are perceived as burdensome. This underscores the importance of designing health promotion content that is not only educational but also entertaining and seamlessly integrated into adolescents’ daily social media use. This aligns with the findings suggesting that gamification plays a crucial role in driving behavior change [45].

While this study provides robust evidence for the preferences and motivations of adolescents regarding Instagram use, alternative explanations should be considered. For example, the preference for quizzes and polls might reflect a broader trend toward gamification in digital environments rather than an inherent preference for these specific features. Future research should explore these possibilities to ensure a comprehensive understanding of adolescents’ digital behavior.

### Strengths and Limitations

One of the primary strengths of this study is its qualitative research design, which allowed for an in-depth exploration of adolescents’ preferences, attitudes, and motivations regarding the use of Instagram for health promotion. The semistructured interviews and focus groups in this study facilitated open-ended discussions, enabling participants to express their thoughts freely and providing the research team with a deep understanding of the nuances and contextual factors that influence adolescents’ engagement with digital health interventions [87]. Another strength of this study is the iterative refinement of the coding system used in the content analysis. By starting with a deductive approach based on existing literature and then refining the categories through an inductive process, this study was able to develop a robust and comprehensive framework for analyzing the data. The high intercoder reliability achieved (Cohen κ=0.93) further underscores the methodological rigor and consistency of the analysis [96]. This process ensured that the findings were grounded in both theoretical knowledge and empirical data, enhancing the credibility and validity of the study.

Despite its strengths, the study also has several limitations that need to be acknowledged. One of the primary challenges of qualitative research is its limited generalizability. Given the in-depth nature of the data collection, qualitative studies typically involve smaller sample sizes, which means that the findings may not be representative of the broader population [97]. In this study, the data were collected from a relatively small group of adolescents (67 participants) in Germany, which, although diverse in educational backgrounds, may not fully capture the range of experiences and perspectives present in the wider adolescent population. First, the generalizability for Germany is already clearly limited, as participants only came from the region surrounding the university and the researchers (Mecklenburg–Western Pomerania and Berlin). In addition, the global generalizability of the findings is limited, as social media use and health perceptions are strongly shaped by regional and cultural differences, such as privacy norms, consumption and distribution patterns, and culturally grounded worldviews [98-100]. Furthermore, adolescents who are more engaged with Instagram or have access to certain educational resources may be overrepresented, while those from different socioeconomic backgrounds or regions may be underrepresented. This limitation suggests that the findings should be interpreted with caution, particularly when considering their applicability or generalizability to other populations. Accordingly, the transferability of these findings across different contexts and countries should be approached with caution. The specific preferences and social media–related behaviors discussed in this study may vary depending on cultural, social, and technological contexts. Future studies should aim to replicate these findings in diverse settings to determine the extent to which they can be generalized.

Another limitation relates to the potential for social desirability bias, which is a common issue in qualitative research where participants may provide responses they believe are expected or socially acceptable rather than their true thoughts and feelings [101]. While the research team took steps to create an open and nonjudgmental environment during the interviews and focus groups, it is possible that some participants may have downplayed negative aspects of social media use or overemphasized their conviction of the possibilities of health promotion on Instagram.

While we intended to foster a safe and nonjudgmental environment, which was crucial for encouraging participants to openly share their thoughts, this supportive atmosphere may have inadvertently influenced some responses. Although this approach was intentional to reduce anxiety and promote honest dialogue, it carries the risk of subtly shaping participants’ perceptions. To mitigate this, we aimed for greater objectivity when exploring participants’ social media use, platform features, and thoughts about health promotion strategies. By using open-ended questions, maintaining a neutral stance, and encouraging diverse viewpoints, we aimed to minimize interviewer bias in these discussions. Nevertheless, the interplay between fostering openness and ensuring neutrality remains a methodological challenge, which should be considered when interpreting the findings. Furthermore, the presence of a teacher during the class settings might have further influenced adolescents’ statements and behavior.

Moreover, we did not collect data on participants’ age and gender directly but instead categorized them by school grades, indirectly reflecting the age. However, stratifying groups based on variables, such as gender, education level, or income, in future research could provide valuable insights into differences across these indicators, enabling a deeper exploration of outcome variability and informing more tailored interventions.

The methodological integrity of this study, while generally strong, also faces challenges due to the lack of member checking. Member checking, the process of returning transcripts or findings to participants for validation, can enhance the accuracy and credibility of qualitative research [102]. Due to logistical constraints, this step was not included in the research process, which may limit the verification of the interpretations made by the researchers. Although the high intercoder reliability provides some reassurance regarding the consistency of the coding process, the absence of member checking means that the participants’ perspectives were not directly confirmed in the final analysis. Finally, while the reflexivity of the research team was strongly considered in this study, it is impossible to completely eliminate the impact of the researchers’ perspectives on the study [103]. This is a common challenge in qualitative research where the subjectivity of both the researchers and the participants plays a central role in shaping the findings.

### Implications for Future Research, Policy, and Practice

This study’s findings present various implications for research, policy, and practice. First, the need for further research on how specific Instagram features, such as quizzes, polls, and stories, influence adolescent engagement and behavior change in health promotion was highlighted. Future studies should explore these features across various health domains, such as mental health and substance abuse prevention, as well as their long-term impact on health outcomes. Research should also focus on expanding participant diversity to ensure broader applicability, addressing gaps in understanding cultural and social factors influencing engagement, as suggested by Resnicow et al [74]. In addition, the lack of motivation and the impression of additional workload, identified as barriers to engagement, require deeper investigation to ensure that well-designed interventions reach the target group.

Second, this study underscores the importance of supporting digital literacy initiatives among adolescents, fostering critical thinking, and ensuring they can navigate social media platforms such as Instagram effectively. Policies should also emphasize robust data privacy protections addressing adolescents’ concerns about visibility and anonymity in their online interactions. Policy makers should work toward developing national guidelines for digital health promotion to ensure that interventions are evidence-based and culturally sensitive. Future researchers should continue to prioritize ethical considerations, particularly in ensuring that digital interventions do not inadvertently expose adolescents to new risks.

Third, practitioners should prioritize the use of engaging, interactive content, such as quizzes, polls, and stories, in health promotion programs to capture adolescents’ attention, as supported by the SMUG theory [73]. This study also highlights the importance of simplicity in digital interventions, emphasizing the need for user-friendly designs that minimize barriers to participation. In addition, ensuring cultural sensitivity by using relatable language and imagery is crucial for resonating with diverse adolescent populations. Ethical considerations, particularly regarding privacy and social media–related risks, must be a key focus for practitioners, who should clearly communicate data protection measures to build trust and encourage engagement and further raise awareness of the risks associated with the use of social media. By addressing these implications, future research, policy, and practice can enhance the effectiveness of Instagram and similar digital platforms in promoting adolescent health, leveraging their unique strengths to achieve better health outcomes.

### Conclusions

This study offers significant contributions to the understanding of how Instagram can be effectively used as a platform for health promotion among adolescents. By focusing on the preferences, motivations, and barriers identified by adolescents themselves, this research provides valuable insights that advance the field of digital health interventions. The central contributions of this study lie in its detailed exploration of the specific Instagram features that resonate most with adolescents, such as quizzes, polls, and stories, which have been assessed to enhance engagement and potential behavior change. These findings underscore the importance of interactivity in digital health promotion, aligning with existing theories. By highlighting the need for simplicity, cultural sensitivity, and privacy protections, this study not only supports existing literature but also expands it by addressing gaps related to the unique preferences of adolescents on social media platforms. Furthermore, the implications for future research, policy, and practice underscore the broader impact of these findings. The study’s emphasis on tailored, culturally sensitive interventions that align with adolescents’ digital habits and preferences provides a road map for developing more effective health promotion strategies. This work contributes to a deeper understanding of how to engage adolescents in health promotion efforts meaningfully, offering practical guidelines for leveraging social media platforms such as Instagram in ways that are both effective and respectful of the users’ needs and concerns. In conclusion, this research highlights the potential of Instagram as a powerful tool for adolescent health promotion, providing a foundation for future studies and practical applications that can significantly enhance the well-being of young people. By integrating these findings into broader public health strategies, stakeholders can create more engaging, effective, and accessible health interventions that resonate with the digital realities of today’s adolescents, ultimately contributing to better health outcomes on a larger scale.

## Appendix

Multimedia Appendix 1 Coding system for the qualitative content analysis.

## Abbreviations

SMUG: social media uses and gratifications

## Data Availability

The datasets and research materials generated or analyzed during this study (interviews, codebook, etc) are available in the online repository Open Science Framework [104].
